# The Role of Executive Functions for Motor Performance in Preschool Children as Compared to Young Adults

**DOI:** 10.3389/fpsyg.2020.01552

**Published:** 2020-07-07

**Authors:** Christina Stuhr, Charmayne M. L. Hughes, Tino Stöckel

**Affiliations:** ^1^Sport & Exercise Psychology Unit, Department of Sport Science, University of Rostock, Rostock, Germany; ^2^Health Equity Institute, San Francisco, CA, United States; ^3^Department of Kinesiology, San Francisco State University, San Francisco, CA, United States

**Keywords:** motor–cognition interaction, motor performance, working memory, inhibition, executive functions, child development

## Abstract

Evidence suggests that executive and motor functions are functionally intertwined, with the interrelation between the two processes influenced by the developmental stage of the individual. This study examined executive and motor functions in preschool children (*n* = 41; 65–83 months), and investigated if, and how, preschoolers cognitive–motor functioning differs from that of young adults (*n* = 40; 18–31 years), who served as a control group reflecting the upper bound of cognitive–motor development. As expected, performance of young adults was significantly better than that of preschool children for all cognitive and motor domains tested. The results further showed differential associations among, and between, cognitive and motor functions in preschool children when compared to young adults. While similar correlations among motor variables are found in both groups, correlations among executive functions and between executive and motor variables are only found in preschool children. It thus appears that executive functions (especially working memory) contribute more to successful motor performance in preschool years than in young adulthood. The findings highlight the importance of considering the developmental stage and/or the proficiency level of the individual when examining cognitive–motor interactions or when drawing implications for childhood cognitive–motor training and interventions.

## Introduction

One of the critical determinants of school readiness and academic success is a child’s executive functioning (EF; Blair and Razza, 2007; Borella et al., 2010; Vandenbroucke et al., 2017; Duncan et al., 2018; Korucu et al., 2020), a group of top-down mental processes accountable for goal directed behavior. EFs are comprised of the three key processes: working memory (the temporary storage and manipulation of information in mind; Baddeley and Hitch, 1994), inhibitory control (the regulation of attention, motivation, thoughts and behavior) and cognitive flexibility (the ability of changing perspectives), and higher-level EFs (e.g., response planning and decision making; Diamond, 2013).

Performance on EF tasks is related to the maturation of the prefrontal cortex, as well as other brain regions and connections (e.g., parietal, temporal, or hippocampal areas; Stuss and Benson, 1984; Diamond, 2000; Andrés, 2003). Equivalent to the maturation of the prefrontal cortex (Thompson and Nelson, 2001), EFs exhibit a protracted inverted U-shaped developmental trajectory, with marked increases from early childhood through early adulthood, and subsequent decreases in older adults (De Luca et al., 2003; Lyons-Warren et al., 2004; Zelazo et al., 2004; Diamond, 2013). Similar to the developmental trajectories of EFs, the general functional capacity (Kalache and Kickbusch, 1997) to perform various motor skills follows a protracted maturation across the life span, with peak performance occurring during early adulthood (Clark and Metcalfe, 2002; Leversen et al., 2012; Payne and Isaacs, 2012; Sigmundsson et al., 2016).

The similar developmental trajectories between executive and motor functioning has led researchers to assume a mutual interrelation between the two domains across the lifespan. While there is convincing evidence for a link between the two domains (Livesey et al., 2006; Stöckel and Hughes, 2016; Oberer et al., 2017; Stöckel et al., 2017; Ludyga et al., 2018; Stuhr et al., 2018; van der Fels et al., 2019), research on whether the link differs between a developing cohort and young adults (representing the upper bound of cognitive–motor development) is scarce. Based on the assumption that executive capabilities used to solve more complex tasks change over the course of development (i.e., with well-trained EFs being replaced by newly developed EFs in order to allocate more effort in shaping these newer skills, Best et al., 2009), it is very likely that interrelations between functions differ depending upon the age and ability level of the population under investigation. For example, manual dexterity has been found to be associated with working memory and/or inhibitory control in 5- to 6-year-old children (Livesey et al., 2006; Stöckel and Hughes, 2016; Oberer et al., 2017), and with response planning and cognitive flexibility in young (Stöckel et al., 2017; Stuhr et al., 2018) and older adults (Stöckel et al., 2017). Moreover, gross motor skills have been linked to working memory and inhibitory control in 8- to 10-year-old children (van der Fels et al., 2019), to working memory in adolescents (Rigoli et al., 2012), and to inhibitory control and cognitive flexibility in 6- to 7-year-old children (Roebers and Kauer, 2009; Oberer et al., 2017) and young adult populations (Stuhr et al., 2018).

Interestingly, previous research has indicated that while interrelations between cognitive and motor functions found in young adults appear rather specific (but less strong), the connection between the two domains seems to be stronger at the extreme ends of the lifespan (Livesey et al., 2006; Stöckel and Hughes, 2016; Oberer et al., 2017; Spedden et al., 2017; Stöckel et al., 2017; van der Fels et al., 2019). As such, a first aim of the present study was to assess if, and how, the cognitive–motor functions of preschoolers differ from that of young adults, who served as a control group representing the upper bound of cognitive–motor development. Based on the existing corpus of literature, it is hypothesized that cognitive–motor functioning will be poorer in preschool children as compared with young adults, regardless of the specific domain being tested (Clark and Metcalfe, 2002; De Luca et al., 2003; Leversen et al., 2012; Diamond, 2013). In line with previous work (Clark and Metcalfe, 2002; Diamond, 2013 for a review), we expected that the difference between preschool children and young adults would be greatest for more complex executive and motor functions (e.g., manual dexterity and cognitive flexibility), as these are said to require proficiency in fundamental skills.

A second aim of the study was to explore the specific links between executive and motor functions in preschool children, and to investigate whether these are different from young adults. We hypothesized that executive control processes play a pivotal role for successful motor performance in preschool children (but not in young adults) as most of the motor tasks are challenging and new for the children, and therefore require more cognitive control (Diamond, 2000; Stuhr et al., 2018). However, given that EFs are not fully developed in preschool children (Lee et al., 2013; van der Ven et al., 2013; Monette et al., 2015) we also hypothesized that only the more early-developed core EFs (e.g., working memory, inhibitory control) would be linked to successful motor performance in preschool children (Senn et al., 2004; Livesey et al., 2006; Best et al., 2009; Shing et al., 2010; Stöckel and Hughes, 2016). In contrast, in young adults we expected that the associations between executive and motor functions would be weaker (if existent at all) than those observed in preschool children (Spedden et al., 2017), as cognitive–motor performance is likely automatized and does not require any top-down control in this age group.

## Materials and Methods

### Sample

Forty one 5- to 6-year-old children (age range = 65–83 months, mean age = 71.9 ± 3.9 months, 18 males) and forty young adults (age range = 18–31 years, mean age = 22.1 ± 3.5 years, 25 males) participated in this study. All children were tested approximately half a year before their transition to primary school, and testing occurred in their familiar kindergarten environment. To keep children motivated through all the testing sessions, children were rewarded for their participation after completing all tests. At the start of the first testing day they received a treasure map, were awarded stamps for each test they had completed, and subsequently received a small gift after collecting all stamps on the treasure map. The sample of young adults consisted of undergraduate and graduate university students who received course credit for their participation. To ensure comprehension of all test instructions, participants had to have acquired advanced German language skills that correspond to the fifth level (C1) on the six-level scale of competence laid down in the Common European Framework of Reference for Languages (CEFR). All participants were free from any neurological or mental disorders (e.g., attention deficit hyperactivity disorder) and had normal or corrected to normal vision. The research was approved by the local authorities and the institutional review board, and informed consent and assent was obtained prior to participation.

### Measures and Procedure

The motor performance processes tested were: strength [*Bruininks-Oseretsky Test of Motor Proficiency* (BOT-2)], speed and agility (*BOT-2*), manual dexterity (*Purdue Pegboard test*), and balance [*Star Excursion Balance test* (SEBT)]. The cognitive functions tested were: working memory [*List-Sorting test* (LS)], processing speed [*Simple Reaction Time task* (SRT)], response inhibition [*Hearts and Flowers task* (HF_RT–diff_)], selective attention [*Flanker task* (FF_acc_)], and cognitive flexibility [*Wisconsin Card Sorting task* (WCST_percentcorrect_)].

The SRT, HF_RT–diff_, and FF_acc_ were run using Presentation^®^ (Neurobehavioral Systems Inc., Berkeley, CA, United States) utilizing a 23″ touchscreen monitor (Philips 231C5TJKFU/00) with a custom-built 40 cm long handlebar 5 cm in front of the monitor, on which participants’ hands remained during testing. The remaining cognitive processes were tested using the Psychology Experiment Building Language (PEBL, v0.14; Mueller and Piper, 2014) run on a 12″ Tablet (Samsung Galaxy TabPro S). For the group of young adults, all tests were administered in a single session (but four different rooms) that lasted 2 h. In contrast, children completed the experiment across four consecutive weekdays, with each session lasting between 20 and 30 min each. Test order was counterbalanced across participants.

#### Motor Functioning

The BOT-2 (Blank et al., 2014; Bruininks, 2005) was used to assess the gross motor abilities *strength* and *speed and agility*. Strength was measured using the following subtests: standing long jump (distance measured in cm), knee push-ups and sit-ups (number of accomplished push-ups or sit-ups in 30 s), wall sit and the v-up (time in seconds). Speed and agility was measured using the 30 m shuttle run (time in seconds), stepping sideways over a balance beam, one-legged stationary hop, one-legged side hop and two-legged side hop (number of successful jumps or hops in 15 s). Following the sequence of testing recommended by the BOT-2 testing manual, the speed and agility subtest preceded the strength subtest, with the order of tasks within each subtask following the order outlined in the testing manual. Exploratory factor analysis was used to derive one factor each from the five strength (**BOT**_strength_) and the five speed and agility subtests (**BOT**_speed_), which were then used as the primary outcome measure for strength and speed, respectively.

The *Purdue Pegboard Test* (#32020, Lafayette Instruments, IN, United States) was used to assess *manual dexterity* (Tiffin and Asher, 1948). The Purdue Pegboard consists of a board with two vertical rows of 25 holes each and four concave cups located at the top end of the board that held different items (i.e., pins, washers, collars). Following the standard test protocol (Tiffin and Asher, 1948), participants were asked to insert as many pins into the holes on the board as possible (starting from the top of the board), in a 30 s time period. Participants first performed the task with their right hand, then their left hand, and lastly both hands. To ensure that participants understood the task instructions, each condition started with a practice trial, in which they inserted five pins into the respective holes. The number of pins (or pairs of pins) inserted was averaged across the three trials per condition and summed up for right hand, left hand and both hand conditions. The resulting score (originally referred to as gross manual dexterity score) was used as measure of manual dexterity (**PP**_gross_).

*Balance* was assessed using the modified SEBT (Gray, 1995; Hertel et al., 2006). In this task, participants stood on one leg in the middle of a y-shaped testing grid that had lines extending in the anterior, as well as posterolateral left and right directions. Participants balanced their body on one leg and both hands on the hips, and reached with their other leg in one of the three directions (anterior, posterolateral left, posterolateral right) as far as possible, so that the great toe of the reaching foot made a light touch on the gridline. Instructions emphasized that participants must maintain their weight on the stance leg, that movements be performed directly along the gridline of the corresponding reaching direction, hands should remain on the hips during the entire trial, and to bring both feet back to the start position at the end of each trial. Prior to the test, participants performed four practice trials to ensure they understood the task instructions (Robinson and Gribble, 2008; Munro and Herrington, 2010). Participants performed each condition three times, with reaching direction (anterior, posterolateral left, posterolateral right) and reaching leg (dominant, non-dominant), counterbalanced across participants. The maximum distance (in cm), controlled for the length of participants’ right and left legs (averaged across trials and conditions), was used as primary outcome measure (**SEBT**) for balance control.

#### Executive Functioning

*Working memory* was assessed using a modified *Toolbox List Sorting Working Memory Test* (Tulsky et al., 2014, 2013). In the one-list condition, participants were presented with a series of stimuli from a single category (i.e., either animals [e.g., elephant, cat, mouse, pig] or food [e.g., cherry, hamburger, apple, strawberry]) on a computer monitor for 2 s while the experimenter concurrently verbally stated the name of the stimulus. Participants were asked to remember and recall the items, beginning with the smallest and ending with the largest. The test started with two items and increased by one item after each successful trial. Participants subsequently completed the two-list condition. The procedure was identical to the one-list condition except that items from two categories (i.e., animals and food) were presented. Participants had to order the stimuli from the smallest to the largest items, beginning with all items from the food category, followed by items from the animal category. For each trial, participants received two points if they succeeded on the first attempt and one point if they succeeded on the second attempt. The total number of points achieved in both conditions was used as primary outcome measure **(LS)**.

*Processing speed* was assessed using a *Simple Reaction Time task* (cf. Kiselev et al., 2009). At the start of each trial, a fixation cross was presented for 500 ms, and after a random interval (500–2500 ms) the stimulus (i.e., a red dinosaur) appeared in the middle of the monitor. Participants were asked to respond as quickly as possible to the stimulus by pressing the left mouse button with their right index finger. Trials faster than 100 ms (anticipation errors; Welford, 1980) and slower than two standard deviations above the individual mean (delay errors) were excluded from analysis. The mean reaction time averaged across 32 trials (**SRT**) was used as the primary outcome measure of processing speed.

*Response inhibition* was assessed using the *Hearts and Flowers Test* (Diamond et al., 2007; Wright and Diamond, 2014). Each trial started with a fixation cross presented in the middle of the screen, and after a 500 ms interstimulus interval, the stimuli were then presented for 750 ms. In the first block of 12 trials (congruent condition), a red heart appeared on either the left or the right side of the screen and participants had to touch a button on the same side as the stimuli as fast as possible. In a second block of 12 trials (incongruent condition), a red flower lit up on the screen and participants had to touch the button on the opposite side of the stimuli as fast as possible. For each condition, the average reaction times of the successful trials and response accuracy (rate of correct responses) were computed. Reaction times faster than 250 ms and slower than two standard deviations above the individuals mean were excluded. The primary outcome measure was the difference in mean reaction time (**HF**_RT–diff_) between congruent and incongruent conditions.

*Selective attention* was assessed using the *Flanker task* (Zaitchik et al., 2014), with the stimuli consisting of fish rather than arrows. At the start of each trial, a fixation cross was presented in the middle of the computer monitor for a period of 500 ms, after which five fish arranged in a line were shown for 1500 ms. Participants were asked to press the left or the right button on the touchscreen as fast as possible, depending on the direction the middle fish was facing. In the congruent condition, the middle fish and the outside fish faced the same direction. In the incongruent condition, the middle and outside fish faced different directions. In the no-distraction condition, the middle fish was displayed on its own (i.e., without the flanking fish present), and in a neutral condition the outer fish pointed in an irrelevant direction (i.e., up or down). The inter-trial interval varied randomly between 600 ms and 900 ms. Participants performed 65 trials in total, with 13–17 trials per condition as determined by a random trial generator. Trials faster than 250 ms and slower than two standard derivations above the individual mean were excluded from analysis. The number of correct trials across conditions (in percent) was selected as the primary outcome measure (**FF**_acc_), as it appears to be a sufficiently sensitive measure of selective attention in both children and young adults.

The computerized version of the *Wisconsin Card Sorting test (WCST)* was used to assess *cognitive flexibility* (Grant and Berg, 1948; Welsh et al., 1991; Heaton et al., 1993; Greve, 2001). Participants were asked to sort a series of stimulus cards (64 cards for children, 128 cards for adults) into one of four piles by matching the color (i.e., red, green, blue, yellow), shape (i.e., circle, star, triangle, cross), or number of symbols on the card (i.e., one, two, three, four). Participants were not informed about the classification rule at the start of the task, but received feedback (“correct” or “incorrect”) after each attempt informing them whether the sorting of the respective card matched the present rule. Pilot data indicated that preschool children were unable to fully comprehend written feedback, and were provided with verbal feedback after each trial. In contrast, adults preferred to receive written feedback, and as such post-trial feedback was displayed in written form. After ten consecutive cards had been sorted correctly, the rule changed without prior notice. There was no time limit to sort each card or finish the test. The percentage of correct trials **(WCST**_percentcorrect_) was used as the primary outcome measure of participants’ ability to flexibly adapt to a new rule and give up an old rule.

### Data Processing and Statistical Analyses

Statistical analyses were run on data of eighty-one subjects, with the exception of the WCST (four children did not finish the task) and the Hearts and Flowers Test (data from one child was incomplete). Multiple imputation was used to replace missing values in the study measures. A sequential regression multivariate imputation algorithm was implemented using the *mice* package for *R*. Using Rubin’s rules (Rubin and Schenker, 1991), 15 imputed data sets were recreated using predictive mean matching, with results from the 15 analyses (logistic regression coefficients, confidence intervals [CIs], *P* values) combined to produce the findings reported here.

Two of the forty participants in the young adult group were non-native German speakers. Data was collapsed across mother tongue (native German speakers vs. non-native German speakers) as preliminary data analysis did not reveal any numerical differences due to mother tongue for any of the measured cognitive and motor variables. Consistent with the ubiquitous trend in the field of psychology (cf. Micceri, 1989), our data was non-normal. As such, the data were first transformed by applying a rank-based inverse normal (RIN) transformation, as this has been shown to be effective in transforming skewed data into comparatively more normal data while at the same time minimizing Type I and II error rates (Bishara and Hittner, 2012, 2015).

In the first step of statistical analysis, independent *t*-tests were conducted on the normalized data (separately for each dependent variable) to confirm the substantial differences in motor and EF between preschool children and young adults reported in previous studies. We also quantified the effect size magnitude using the thresholds defined in Cohen (1992): |*d*| < 0.2 were classified as negligible, |*d*| < 0.5 were classified as small, |*d*| < 0.8 were classified as medium, and |*d*| > 0.8 were classified as large. Subsequently, to obtain a more detailed picture regarding the specific relations between cognitive and motor function measures, partial correlations (controlled for age) were performed between all normalized dependent variables, separately for each group. Last, hierarchical multiple regression analyses were used to identify the motor and cognitive variables that predict motor skill behavior, separately for each group. To control statistically for the possible within-group effects of age, we entered age first into all of the regression equations, followed by any motor and cognitive dependent variables found to have significant correlations with each of the four motor domains (strength, speed, manual dexterity, balance).

## Results

### Age-Related Differences in Cognitive and Motor Performance

**FIGURE 1:**
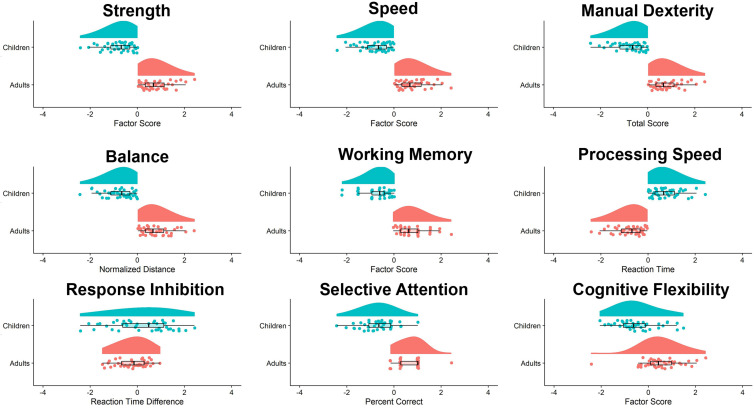
Raincloud plots showing the normalized data, normalized data distribution, and five summary statistics (i.e., normalized median, first quartile, third quartile, minimum, and maximum) for the preschool children (blue) and young adults (red) for the cognitive and motor variables of interest.

Raw means and standard deviations for all motor skill and cognitive measures are displayed in Table 1, while raincloud plots depicting normalized data, normalized data distribution, and five summary statistics (i.e., normalized median, first quartile, third quartile, min, and max) for all metrics are depicted in Figure 1.

**TABLE 1:** Demographic characteristics, descriptive statistics on raw data (means and standard deviation [in parentheses]), as well as results of independent *t*-tests (on the normalized data) used to examine differences in motor and cognitive functioning between preschool children and young adults.

	Children (*n* = 41)	Adults (*n* = 40)	*P*	*T*	Cohen’s *d*
Males, *n* (%)	18 (43.9)	25 (62.5)	–	–	–
Age, years	5.99 (0.32)	22.10 (3.54)	<0.001	840.34	–
Weight, kg	21.62 (3.12)	72.35 (11.62)	<0.001	728.39	–
Height, cm	118.33 (5.69)	178.43 (8.70)	<0.001	1360.30	–
BMI, kg/m^2^	15.43 (1.92)	22.61 (2.41)	<0.001	220.46	–
**Motor**					
Strength (BOT_strength_), standardized factor score	−0.89(0.35)	0.92 (0.48)	<0.001	12.028	2.673
Speed (BOT_speed_), standardized factor score	−0.90(0.44)	0.92 (0.38)	<0.001	12.025	2.672
Manual dexterity (PPgross), number of pins	25.99 (4.10)	44.24 (5.33)	<0.001	–12.047	–2.677
Balance (SEBT), cm	66.8 (14.66)	84.2 (5.32)	<0.001	12.038	2.675
**Cognitive**					
Working memory (LS), points	7.27 (2.50)	17.88 (2.21)	<0.001	12.233	2.719
Processing speed (SRT), reaction time in ms	633.74 (184.99)	252.87 (29.61)	<0.001	–12.022	–2.672
Response inhibition (HF_RT–diff_), reaction time in ms	107.14 (172.99)	30.45 (40.02)	0.962	–1.802	–0.398
Selective attention (FF_acc_), accuracy in%	60.4 (21.6)	90.9 (4.5)	<0.001	10.326	2.287
Cognitive flexibility (WCST_percentcorrect_), accuracy in%	55.32 (21.51)	80.52 (6.36)	<0.001	5.398	1.199

As can be seen in Table 1, young adults outperformed preschool children for all tested motor variables (all *p*’s < 0.001), with large effect sizes observed (*d* range: |2.672| – |2.677|). A similar pattern emerged for the cognitive variables. With the exception of response inhibition (*p* = 0.962), children had particularly low scores compared to the young adult group (all *p*’s < 0.001). Large effect sizes were observed for working memory, processing speed, selective attention (all *d*’s > |2.287|) and cognitive flexibility (*d* = |1.199|). In contrast, the effect size for response inhibition was small (*d* = |0.398|).

### Age-Related Differences in Task-Specific Associations Between Cognitive and Motor Domains

Follow-up correlation analyses provided a more detailed picture about how the specific associations between executive and motor functions in preschool children and young adults differ. Correlational analysis for preschool children are shown in the upper triangle of Table 2. In preschool children, all of the tested motor skills were positively correlated with one another (*r* range: |0.381| – |0.591|, all *p*’s < 0.01). There were also correlations observed between the measured cognitive variables, and between the cognitive and motor variables. Specifically, working memory was correlated with selective attention and cognitive flexibility (*r* = 0.340 and 0.411, respectively, both *p*’s < 0.001), as well as all of the tested motor skills (i.e., strength: *r* = 0.559, speed: *r* = 0.501, manual dexterity: *r* = 0.718) expect balance (*r* = 0.301, *p* = 0.059). Processing speed was negatively correlated with the motor function speed (*r* = −0.355, *p* = 0.020), indicating that faster processing speed (indicative of better performance) is associated with higher factor scores on the speed task (indicative of better performance). Selective attention was correlated with manual dexterity (*r* = 0.370), as well as the cognitive functions working memory (*r* = 0.340) and cognitive flexibility (*r* = 0.448), all *p*’s < 0.05. Cognitive flexibility was not associated with any of the tested motor functions (*r* range: |0.110| – |0.277|, all *p*’s > 0.05), but was positively correlated with working memory and selective attention (*r* = 0.411 and 0.448, respectively, both *p*’s < 0.001). Response inhibition was not correlated with any of the tested motor and cognitive variables (*r* range: |0.021| – |0.241|, all *p*’s > 0.05).

**TABLE 2:** Partial correlations between the motor skill components (strength, speed, manual dexterity, balance) and measures of executive functioning (working memory, processing speed, response inhibition, selective attention, cognitive flexibility) in preschool children (upper triangle, gray shaded) and young adults (lower triangle).

	BOT_strength_	BOT_speed_	PPgross	SEBT	LS	SRT	HF_RT–diff_	FFacc	WCST_percentcorrect_
**BOT_strength_**		**0.591**	**0.590**	**0.433**	**0.559**	−0.252	0.070	0.184	0.159
**BOT_speed_**	**0.698**		**0.381**	**0.514**	**0.501**	−**0.355**	−0.035	0.197	0.110
**PPgross**	−**0.347**	−0.298		**0.519**	**0.718**	−0.208	0.047	**0.370**	0.277
**SEBT**	**0.549**	**0.440**	−**0.404**		0.301	−0.212	−0.021	0.296	0.119
**LS**	−0.137	−0.117	0.158	0.038		−0.164	0.132	**0.340**	**0.411**
**SRT**	−0.295	−0.248	−0.121	−0.017	0.015		−0.023	−0.283	−0.179
**HF_RT–diff_**	−0.250	−0.047	−0.028	−0.146	0.089	−0.063		0.162	0.241
**FFacc**	−0.245	−0.217	−0.033	−0.014	0.105	0.190	−0.169		**0.448**
**WCST_percentcorrect_**	−0.015	−0.029	0.152	0.094	−0.124	−0.008	−0.222	0.282	

*Bold-typed values represent significance, p < 0.05.*

Correlational analysis for young adults are shown in the lower triangle of Table 2. With the exception of the association between speed and manual dexterity (*r* = −0.298, *p* > 0.05), all of the measured motor skills were correlated with one another (*r* range: |0.347| – |0.698|, all *p*’s < 0.01). Specifically, strength was positively correlated with speed and balance (respective *r*’s = 0.698 and 0.549, both *p*’s < 0.05) and negatively correlated with manual dexterity (*r* = −0.347, *p* < 0.05), indicating that higher scores on the strength task were associated with better performance on the balance task, but worse manual dexterity performance. None of the cognitive variables were significantly associated with one another (*r* range: |0.008| – |0.282|, all *p*’s > 0.05), nor were any of the cognitive and motor variables (*r* range: |0.014| – |0.295|, all *p*’s > 0.05).

### Specific Processes Associated With Different Motor Skills

Hierarchical multiple regression analyses was used to identify the motor and cognitive variables that predict motor skill behavior, separately for each group. The results of each step in the regression analysis and individual standardized beta coefficients with associated significance are provided in Table 3 (preschool children) and Table 4 (young adults).

**TABLE 3:** Hierarchical multiple regression results: Children.

Motor skill	Model		Standardized β	*P*	Adjusted *R*^2^	*R*^2^ change	*P*
Strength	Model 1	Age	0.019	0.907	−0.025	0.000	0.907
	Model 2	Age	−0.264	0.077	0.439	0.508	**<0.001**
		Speed	0.480	0.015			
		Manual dexterity	0.404	0.070			
		Balance	0.009	0.957			
		Working memory	0.101	0.628			
Speed	Model 1	Age	0.186	0.243	0.010	0.035	0.243
	Model 2	Age	0.213	0.138	0.487	0.529	**<0.001**
		Strength	0.356	**0.023**			
		Manual dexterity	−0.405	0.058			
		Balance	0.383	**0.010**			
		Working memory	0.443	**0.021**			
		Processing speed	−0.191	0.120			
Manual dexterity	Model 1	Age	0.424	**0.006**	0.159	0.180	**0.006**
	Model 2	Age	0.292	**0.007**	0.682	0.550	**<0.001**
		Strength	0.231	0.066			
		Speed	−0.227	0.077			
		Balance	0.313	**0.008**			
		Working memory	0.555	**<0.001**			
		Selective attention	0.071	0.480			
Balance	Model 1	Age	−0.155	0.333	−0.001	0.024	0.333
	Model 2	Age	−0.404	**0.011**	0.335	0.377	**<0.001**
		Strength	−0.018	0.920			
		Speed	0.381	**0.025**			
		Manual dexterity	0.421	**0.023**			

*Bold-typed values represent significance, p < 0.05.*

**TABLE 4:** Hierarchical multiple regression results: Adults.

Motor Skill	Model		Standardized β	*P*	Adjusted *R*^2^	*R*^2^ change	*P*
Strength	Model 1	Age	0.051	0.754	−0.024	0.003	0.754
	Model 2	Age	0.104	0.361	0.515	0.562	**<0.001**
		Speed	0.556	<0.001			
		Manual dexterity	−0.069	0.579			
		Balance	0.277	**0.043**			
Speed	Model 1	Age	−0.050	0.758	−0.024	0.003	<0.758
	Model 2	Age	−0.075	0.538	0.451	0.491	**<0.001**
		Strength	0.653	**<0.001**			
		Balance	0.082	0.567			
Manual dexterity	Model 1	Age	−0.062	0.705	−0.022	0.004	0.705
	Model 2	Age	−0.086	0.578	0.121	0.185	0.054
		Strength	−0.179	0.327			
		Balance	−0.307	0.097			
Balance	Model 1	Age	−0.107	0.510	−0.015	0.011	0.510
	Model 2	Age	−0.138	0.317	0.291	0.353	**<0.003**
		Strength	0.405	**0.043**			
		Speed	0.085	0.655			
		Manual dexterity	−0.237	0.110			

*Bold-typed values represent significance, p < 0.05.*

With respect to preschool children, correlation analysis revealed that strength was significantly related to speed, manual dexterity, balance, and working memory. Although age alone did not predict strength performance (adjusted *R*^2^ = −0.025, *p* = 0.907), strength was predicted by the full model (adjusted *R*^2^ = 0.439, *p* < 0.001), with speed emerging as a unique contributor of strength performance (β = 0.480, *p* = 0.015). Speed was significantly correlated with strength, manual dexterity, balance, working memory, and processing speed. Regression analysis revealed that while speed could not be significantly predicted by age alone (adjusted *R*^2^ = 0.010, *p* = 0.243), it was predicted by the full model (adjusted *R*^2^ = 0.487, *p* < 0.001), with strength (β = 0.356, *p* = 0.023), balance (β = 0.383, *p* = 0.010), and working memory (β = 0.443, *p* = 0.021) emerging as unique predictors of speed. Manual dexterity performance was significantly correlated with strength, speed, balance, working memory, and selective attention. Regression analysis indicated that manual dexterity was significantly predicted by participant age (adjusted *R*^2^ = 0.159, *p* = 0.006). However, the full model explained more of the variance of manual dexterity performance (adjusted *R*^2^ = 0.682, *p* < 0.001) above and beyond that of model 1 (*R*^2^ change = 0.550). With respect to the full model, age (β = 0.292, *p* = 0.007), balance (β = 0.313, *p* = 0.008), and working memory (β = 0.555, *p* < 0.001) emerged as unique contributors of manual dexterity performance. Balance performance was significantly correlated with strength, speed, and manual dexterity. Regression analysis indicated that balance was not predicted by participant age (adjusted *R*^2^ = −0.001, *p* = 0.333). It was, however, predicted by the full model (adjusted *R*^2^ = 0.335, *p* < 0.001), with age (β = −0.404, *p* = 0.011), speed (β = 0.381, *p* = 0.025), and manual dexterity (β = 0.421, *p* = 0.023) emerging as unique predictors.

In the young adult group, correlational analysis revealed that strength was correlated with speed, manual dexterity, and balance. When entered into the regression analysis, it was found that strength was not predicted by the age-only model (adjusted *R*^2^ = −0.024, *p* = 0.754). It was, however, significantly predicted by the full model (adjusted *R*^2^ = 0.515, *p* < 0.001), with speed and balance emerging as unique contributors of strength performance in young adults (β = 0.556, *p* < 0.001 and β = 0.277, *p* = 0.043, respectively). Speed was significantly correlated with strength and balance. Although age did not predict speed performance (adjusted *R*^2^ = −0.024, *p* = 0.758), regression analysis revealed that the full model predicted speed performance (adjusted *R*^2^ = 0.451, *p* < 0.001), with strength (β = 0.653, *p* < 0.001) emerging as a unique predictor of speed performance in young adults. Manual dexterity of the adult sample was associated with strength and balance performance, however, regression analysis indicated that neither the age-only nor the full model (adjusted *R*^2^ = −0.022, *p* = 0.705 and adjusted *R*^2^ = 0.121, *p* = 0.054, respectively) significantly predicted manual dexterity performance. Young adults balance was associated with all of the tested motor functions. Regression analysis indicated that balance performance was significantly predicted by the full model (adjusted *R*^2^ = 0.291, *p* = 0.003), with strength emerging as a unique predictor (β = 0.405, *p* = 0.043).

## Discussion

A first aim of the present study was to assess if, and how, preschoolers’ cognitive–motor functions differ from that of young adults (i.e., indicative of upper bound performance). Congruent with our hypotheses and prior studies (Clark and Metcalfe, 2002; De Luca et al., 2003; Leversen et al., 2012; Payne and Isaacs, 2012; Diamond, 2013), we found that young adults outperformed children in all but one of the tested cognitive and motor processes (i.e., response inhibition). This is not surprising given that human motor development is marked by both qualitative (e.g., more efficient movement patterns; cf. Motor Development Task Force, 1995) and quantitative progressions in performance (e.g., increases in smoothness, time to peak velocity, improvements in motor planning, cf. Payne and Isaacs, 2012; Stöckel and Hughes, 2015) from early childhood to early adulthood. Similarly, EFs have been found to mature from childhood throughout adolescence and into early adulthood (De Luca et al., 2003; Huizinga et al., 2006), which are said to result from maturation of the frontal lobes and other brain regions (e.g., parietal, temporal, or hippocampal; Andrés, 2003; Casey et al., 2005), as well as increases in cortical white and gray matter (Giedd et al., 1999; Gogtay et al., 2004). Interestingly, there was a negligible overlap in performance between preschoolers and young adults (as depicted by the raincloud plots in Figure 1) for most of the cognitive and motor functions tested, especially strength, speed, manual dexterity, balance, working memory, and processing speed. In contrast, response inhibition performance, selective attention, and cognitive flexibility of some preschool children were as good as that of young adults, indicating that aspects of these functions may develop earlier than the others and serve as fundamental building blocks for other more complex skills (cf. Clark and Metcalfe, 2002; Best et al., 2009).

A second aim of the study was to explore the specific links between motor and executive functions in preschool children, and to investigate whether these are different from young adults. While correlations among motor functions were found for both groups, correlations among executive functions, as well as between motor and executive functions, were found only for the preschool children. Specifically, in preschool children working memory performance was positively associated with strength, speed, and manual dexterity. Additionally, processing speed was associated with speed, and selective attention was correlated with manual dexterity. Further, regression analyses revealed that working memory explained unique portions of speed and manual dexterity in preschool children (see Table 3). Taken together, these results indicate that children with better working memory exhibited better performance during the strength, speed, and manual dexterity tasks, whereas working memory was not associated with motor performance in young adult group. These results are in line with previous studies in preschool children reporting weak to moderate positive associations between manual dexterity and the EFs working memory and inhibitory control (Livesey et al., 2006; Röthlisberger et al., 2010; Stöckel and Hughes, 2016), as well as between (fine and gross) motor skills and the EFs working memory (Roebers et al., 2014; Oberer et al., 2017) and inhibitory control (Oberer et al., 2017).

The emerging picture from this corpus of work is that working memory and motor functions (strength, speed, manual dexterity) are linked in preschool children, although the size of the effect may vary based on the studied sample and the task employed. Although only a few studies have investigated the interplay between motor and cognitive domains in young adults (Spedden et al., 2017; Stöckel et al., 2017; Stuhr et al., 2018), the present results support prior work demonstrating that the two domains have significantly fewer connections (or exhibit a weaker association) in young adults (e.g., Stöckel et al., 2017; Stuhr et al., 2018) than in children (e.g., Stöckel and Hughes, 2016; Oberer et al., 2017) or older adults (Spedden et al., 2017; Stöckel et al., 2017).

Interestingly, results of the present study indicate that of the tested EFs, preschoolers’ response inhibition performance was closest to young adult levels, and was the only EF that was not correlated with any other executive or motor function for either group. Moreover, there was substantial variation in response inhibition performance among the preschool group, which was not observed for the other tested variables. In contrast to these results, working memory was positively correlated with the EFs selective attention, and cognitive flexibility, as well as the motor functions strength, speed, and manual dexterity in preschool children. In this respect, there is agreement that individual EFs exhibit different developmental trajectories during child development (Best and Miller, 2010; Diamond, 2013). That is, the trajectory of working memory exhibits a somewhat linear improvement from preschool through adolescence, while there is a rapid increase in inhibitory control during the preschool period followed by a moderate improvement rate thereafter (Best and Miller, 2010). It can therefore be argued that the rapid improvement in inhibitory control may have already occurred in some of the preschool children in our sample, whereas the steady development of working memory is still underway.

Following the argument that the EFs used to solve more complex tasks change over the course of development (Best et al., 2009), it could further be argued that the developmental period between ages 5- to 6-years is critical for the improvement of working memory, as it appears to play a pivotal role in all cognitive–motor functioning. Interestingly, the importance of working memory for proficient motor functioning appears to be constantly high at least until adolescence (Rigoli et al., 2012; Ludyga et al., 2018; van der Fels et al., 2019), which is consistent with the assumption that working memory performance improves in a linear fashion (Best and Miller, 2010), with a consistent covariance of working memory with other EFs observed until adolescence (Hartung et al., 2020). Based on the above argument, other EFs may be associated to motor performance when working memory is developed to a certain level (i.e., to a proficient level), which facilitates the development of new or more complex EFs, such as cognitive flexibility or planning and problem solving (cf. Best et al., 2009). This assumption is supported by previous studies that have shown associations between motor functions and higher executive functions in young adult populations (Stöckel et al., 2017; Stuhr et al., 2018).

Taken together, our data suggest that (a) EFs contribute more to successful motor performance during preschool years than young adulthood, and that (b) working memory plays an overarching role in the performance of motor and cognitive functioning in preschool children. Indeed, the current data suggests that researchers should be careful generalizing findings regarding the relationship between motor and cognitive functions across different age groups and motor skills. Rather, it appears that the interplay between motor and cognitive domains underlie dynamic and structural changes during child development depending on motor and cognitive proficiency. In line with our finding that EFs are associated to one another in children but not in young adults, several studies have demonstrated that the inner relationship between EFs change over time (Senn et al., 2004; Best et al., 2009; Chevalier et al., 2012; Hartung et al., 2020). There is consensus in the literature that EF performance becomes increasingly differentiated over the course of development (Garrett, 1946; Li et al., 2004; de Frias et al., 2007; Garon et al., 2008; Shing et al., 2010), such that the performance of only one or two EF can be distinguished from one another during early childhood, while the performance of all three EFs are typically distinguishable from one another in adult populations (Miyake et al., 2000; Usai et al., 2014; Monette et al., 2015). In that regard, it seems reasonable that the interplay between motor and cognitive functions is mutable as long as the neural processes associated with ongoing EF development are occurring. That said, it appears that the cognitive–motor link is highly adaptive and influenced by the maturation of EFs from early childhood to early adulthood.

This study provides new evidence about the interaction between motor control and cognition in developing children, and how it differs from young adults (a group representing the upper bound of cognitive–motor functioning). However, there are some limitations to the current study, which may inform future directions in this line of research. First, we only tested a single developmental age group using a cross-sectional design. Given the swift change in sensorimotor and cognitive skills during the preschool and primary school years, the next step in this line of work would be to investigate children across a large span of the developmental spectrum, and analyze the moderating effects of environmental factors (e.g., socioeconomic status, bilingualism, perinatal health) on motor and cognitive functioning. In addition, longitudinal designs with follow-ups over several years are necessary to draw firm conclusions regarding the sensitive periods of sensorimotor and cognitive development necessary to inform the most pertinent ages that EF interventions should occur.

Second, differences in methodology (i.e., the choice of EF and/or motor tasks, selection of outcome variables) influences the extent to which the present findings can be compared to previous work, such that the results of the present study can only be directly compared to prior empirical work that uses similar tasks and outcome variables. However, there is an often unacknowledged benefit in utilizing different measures, such that using a variety of measures and tests to study the same problem may help to generalize findings or detect task-specific effects. In future studies it would certainly be worthwhile to use more than one test to study a single sensorimotor and/or cognitive component (and to report all relevant scores of a single test), as this would increase both the comparability and generalizability of findings. For example, the substantial within-group variation in response inhibition performance among preschoolers may relate to the task or dependent variables used to evaluate response inhibition, or the executive function itself. While we can only speculate on the source of variability based on the results of the present study, the use of multiple measures would help clarify this issue.

Limitations notwithstanding, findings of the present study contribute to the corpus of literature regarding the link between motor control and cognition at different stages in the lifespan, with present results indicating that working memory is involved in most motor skills during early childhood. From an applied perspective, the growing corpus of research in this area indicates interventions combining working memory and sensorimotor training may benefit preschool children with motor delays and/or motor impairments (Lakes and Hoyt, 2004; Diamond, 2012; Diamond and Ling, 2016), more than normally developed preschool children, children in late childhood, adolescents, or young adults.

## Data Availability Statement

The datasets generated for this study are available on request to the corresponding author.

## Ethics Statement

The studies involving human participants were reviewed and approved by the San Francisco State University. Written informed consent to participate in this study was provided by the participants’ legal guardian/next of kin.

## Author Contributions

CS and TS developed the study protocol and contributed equally to data collection. All authors equally contributed to the analysis and interpretation of the results, and to drafting the article. All authors approved the final version.

## Conflict of Interest

The authors declare that the research was conducted in the absence of any commercial or financial relationships that could be construed as a potential conflict of interest.
